# Structure/Properties Relationship of Anionically Synthesized Diblock Copolymers “*Grafted to*” Chemically Modified Graphene

**DOI:** 10.3390/polym13142308

**Published:** 2021-07-14

**Authors:** Dimitrios Katsigiannopoulos, Eftychia Grana, Konstantina Tsitoni, Ioannis Moutsios, Gkreti-Maria Manesi, Evgeniia A. Nikitina, Nikolaos Chalmpes, Dimitrios Moschovas, Dimitrios Gournis, Dimitri A. Ivanov, Apostolos Avgeropoulos

**Affiliations:** 1 Department of Materials Science Engineering, University Campus-Dourouti, University of Ioannina, 45110 Ioannina, Greece; Dimitris.Katsigiannopoulos@gmail.com (D.K.); Eftychia.Grana@gmail.com (E.G.); k.tsitoni@uoi.gr (K.T.); imoutsios@uoi.gr (I.M.); gretimanesi@uoi.gr (G.-M.M.); chalmpesnikos@gmail.com (N.C.); dmoschov@uoi.gr (D.M.); dgourni@uoi.gr (D.G.); 2 Faculty of Chemistry, Lomonosov Moscow State University (MSU), GSP-1, 1-3 Leninskiye Gory, 119991 Moscow, Russia; nikitina.ea@phystech.edu (E.A.N.); dimitri.ivanov.2014@gmail.com (D.A.I.); 3 Institute of Problems of Chemical Physics, Russian Academy of Sciences, Chernogolovka, 142432 Moscow, Russia; 4 Institut de Sciences des Matériaux de Mulhouse—IS2M, CNRS UMR7361, 15 Jean Starcky, 68057 Mulhouse, France

**Keywords:** composite nanomaterials, anionic polymerization, sequential addition of monomers, SEC, ^1^H-NMR, Raman spectroscopy, XRD, TEM

## Abstract

A novel approach to obtaining nanocomposite materials using anionic sequential polymerization and post-synthetic esterification reactions with chemically modified graphene sheets (CMGs) is reported. The anionically synthesized diblock copolymer precursors of the PS-*b*-PI-OH type were grafted to the chemically modified –COOH groups of the CMGs, giving rise to the final composite materials, namely polystyrene-*b*-poly(isoprene)-*g*-CMGs, which exhibited enhanced physicochemical properties. The successful synthesis was determined through multiple molecular characterization techniques together with thermogravimetric analysis for the verification of increased thermal stability, and the structure/properties relationship was justified through transmission electron microscopy. Furthermore, the arrangement of CMGs utilizing lamellar and cylindrical morphologies was studied in order to determine the effect of the loaded CMGs in the adopted topologies.

## 1. Introduction

Graphene oxide (GO) nanosheets constitute a highly investigated topic of research, rendering their use imperative in a multitude of applications, holding exceptional properties such as mechanical strength, thermal, and electric conductivity [1,2,3,4]. The hydrophobic two-dimensional (2-D) sp^2^ carbon nanostructures are susceptible to covalent or non-covalent surface chemical modification, inducing functional groups able to react with polymeric chains [1,5]. Grafting polymers on modified graphene sheets results in nanocomposites presenting a combination of properties resulting from both materials, namely improved solubility and interfacial energy alternations [6,7,8,9,10,11,12,13,14,15,16]. The reaction between polymers and graphene oxide nanosheets involves two methods and specifically the “*grafting from*” [17], where the GO–initiator complex is primarily prepared in order to initiate the polymerization from the GO surface, and the “*grafting to*” [18], where the polymeric precursor bearing a functional group reacts with the chemically modified GO [19,20,21,22,23]. Employing the “*grafting to*” method, better control over the polymer’s molecular characteristics can be realized and satisfactory dispersity, and solubility are achieved, while the “*grafting from*” method results in higher coupling yields, but limited control over the molecular characteristics is reported [24].

Several methods for preparing polymer/graphene composites have been exploited involving the utilization of various polymerization techniques, such as “click” chemistry reactions [25,26,27], atom transfer radical (ATRP) [28,29,30], reversible addition fragmentation chain transfer (RAFT) [31,32,33], and ionic polymerizations [34,35,36], in combination with functionalized graphene groups (carboxyl, hydroxyl, and so on), which are already reported in the literature [37,38].

Although the abovementioned polymerization methods have been extensively studied for the synthesis of various composite materials, resulting in promising applications such as nanoelectronics, sensors [39], water treatment and gas/transport membranes, energy storage, and so on [40], ionic polymerization methods for the preparation of nanocomposite materials are only scarcely adopted [35,36,37].

The highly demanding purifying and synthetic protocols in combination with the limited number of monomers bearing functional side groups on both anionic and cationic polymerization impede the exploitation of these synthetic methods, as the use of GO completely reduces the reactivity of the initiators, resulting in low yields during reaction [39].

Cationic polymerization of vinyl monomers (e.g., vinyl ethers, indene, acenaphthylene) “*grafting from*” or “*grafting to*” GO has been achieved by the non-nucleophilic addition attributed to the intensified acidic character, thanks to the existence of –COOH groups on the modified graphene sheet surface [41,42,43]. Various total number average molecular weights of polyester, polyamide, and poly(caprolactone) composites have been reported by Bielawski et al. [44], utilizing cationic polymerization using GO as catalyst. The use of GO as a cationic initiator has also been expanded for a diblock copolymer system of the polystyrene-*b*-polyisoprene (PS-*b*-PI) type and a PS homopolymer, resulting in composites presenting satisfactory dispersion in many organic solvents. The increased sheet-to-sheet distance exhibited in the aforementioned diblock copolymers gave rise to porous structures, rendering the specific composite materials appealing for membrane applications and specifically for gas permeability and chemical separation [36]. Homopolymer composites of GO-*g*-benzoxazine type have been prepared using GO as cationic initiator and subsequently thermally characterized, leading to enhanced thermal stability [45].

On the other hand, the neutralization of the carboxyl groups (–COOH) through potassium hydroxide (–KOH) led to the formation of potassium carboxylate (–COOK) side groups on the GO, enabling the anionic polymerization of epoxides and cyclic acid anhydrides [46,47].

Herein, we report for the first time, to the best of our knowledge, the preparation of composite materials constituting anionically synthesized diblock copolymers of the PS-*b*-PI-OH type with chemically modified graphene oxide (CMGs) through the “*grafting to*” method. The diblock copolymer precursors were end-capped with two monomeric units of ethylene oxide prior to the polymerization termination, in order to bear functional hydroxyl side groups capable of reacting through esterification reaction with the carboxyl GO groups, eventually obtaining the composite materials, namely PS-*b*-PI-*g*-CMGs. The successful synthesis of the intermediate PS-*b*-PI-OH products was verified through molecular characterizations, such as size exclusion chromatography (SEC), proton nuclear magnetic resonance spectroscopy (^1^H-NMR), and infrared spectroscopy (FT-IR). The thermal characterization through thermogravimetric analysis (TGA) determined the thermal stability of both intermediate and final materials. Raman spectroscopy was used to verify the structure of the carbon nanoforms, while X-ray diffraction (XRD) was employed in order to evaluate possible changes between the spaces of the consecutive layers of the CMGs. Transmission electron microscopy (TEM) experiments were also conducted in order to study structure/properties relationship after the incorporation of the CMGs in the corresponding polymer matrices, as well as the successful composite formation. The inability of graphitic structures to be dispersed in common organic solvents for long periods was compensated after the CMGs were covalently bonded to the diblock copolymers, leading to the observation of enhanced dispersion in organic solvents for significantly longer periods.

## 2. Materials and Methods

### 2.1. Materials

Monomers (styrene, isoprene, and ethylene oxide), initiators (*n*-BuLi, *sec*-BuLi), and solvents (benzene, tetrahydrofuran (THF), and methanol), as well as nitric acid (HNO_3,_ 70%), sulfuric acid (H_2_SO_4,_ 97%), potassium chlorate (KClO_3,_ 99%), sodium hydroxide (NaOH, 97%), hydrochloric acid (HCl, 37%), *N,N*′-dicyclohexylcarbodiimide (DCC), 4-dimethylaminopyridine (DMAP), and powdered graphite (powder, <20 μm), employed for the functionalization of the GO, were purchased from Sigma-Aldrich, St. Louis, MO, USA. All reagents used for the anionic polymerization were purified as already thoroughly described in the literature [48,49], while compounds concerning GO were used without further purification. The Staudenmaier method [5] was employed in order to receive exfoliated graphene oxide sheets.

### 2.2. Methods

The molecular characterization was accomplished through size exclusion chromatography (SEC) utilizing a PL-GPC 50 Integrated GPC System from Agilent Technologies (St. Clara, CA, USA) and was calibrated with ten PS standards M_p_: 1.2 kg/mol to 1500 kg/mol. The eluent utilized by the specific technique was tetrahydrofuran (THF) with a flow rate of 1.0 mL min^−1^.

^1^H-NMR spectroscopy (Bruker GmbH, Berlin, Germany) was employed in order to determine the mass and volume fractions of each segment as well as to verify the successful end-capping reaction of the poly(isoprene) with the –OH groups. The spectra were obtained at room temperature in CDCl_3_ on a Bruker AV-400 Avance using a frequency of 400 MHz. Data were processed using UXNMR (Bruker) software.

Infrared spectroscopy (FT-IR) was carried out using a FTIR spectrometer JASCO FT/IR (JASCO, Easton, MD, USA). Spectra were retrieved by employing 32 scans ranging from 4000 to 400 cm^−1^ with a resolution of 2 cm^−1^ under ambient conditions.

Raman spectroscopy (RS) was realized via micro-Raman RM 1000 Renishaw system (Renishaw, Wotton-under-Edge, UK). The power of the laser was 30 mW and a 2 μm focus spot was used.

Thermogravimetric analysis (TGA) was performed using a Perkin Elmer Pyris-Diamond instrument (PerkinElmer, Inc., Waltham, MA, USA). Approximately 10 mg of the sample was heated from 40 °C to 700 °C following a step of 5 °C/min.

Transmission electron microscopy (TEM) was conducted with a JEOL 2100 TEM (JEOL Ltd., Tokyo, Japan) using 200 KeV as the acceleration voltage. Cryo-ultramicrotoming of the films was realized in a Leica EM UC7 ultramicrotome (Leica EM UC7 from Leica Microsystems, Wetzlar, Germany) below the glass transition temperature of both blocks (−100 °C) in order to obtain very thin sections (~30 nm) and the sections were picked up on 400 mesh copper grids.

X-ray diffraction (XRD) measurements were carried out in a Bruker D8 Advance (Bruker, Billerica, MA, USA) with Bragg–Brentano geometry with LYNXEYE detector in 2θ range from 2° to 30°. For the X-rays, a Cu-K_α_ wire was used, resulting in a radiation of 1.5406 Å wavelength.

### 2.3. Synthesis of PS-b-PI-OH

The diblock copolymers of the PS-*b*-PI-OH type were synthesized via anionic polymerization under high vacuum using sequential monomer addition (Scheme S1a, Supplementary Materials). The synthesis of the intermediate product (e.g., 1-PS-*b*-PI-OH) was accomplished after the polymerization of the PS segment using styrene (5 g, 0.05 mol) and *sec*-BuLi (0.24 mmol) in the presence of a non-polar solvent (benzene, 300 mL). Afterwards, the PI segment was synthesized sequentially by introducing isoprene (4.2 g, 0.06 mol) in the living PS macroinitiator and, eventually, the living diblock copolymer was end-capped with two monomeric units of ethylene oxide (0.72 mmol) prior to termination with degassed methanol.

### 2.4. Synthesis of Carboxylated Graphene Oxide (CMGs)

The carboxylation of graphene oxide sheets was accomplished through the already established method by Park et al. [50] GO sheets were dissolved in dionized water (~4 mg/mL) and ultra-sonicated for 1 h before an equal quantity of NaOH (3M) was added to the solution and further sonicated for 3 h. Finally, HCl was added and, after the complete neutralization of the solution, the final solid product (GO–COOH) was filtered and rinsed (Scheme S1b, Supplementary Materials).

### 2.5. Synthesis of PS-b-PI-g-CMGs

In a round-bottom flask under inert atmosphere containing 250 mL of anhydrous THF, 1.5 g of each diblock copolymer precursor together with 0.150 g or 0.450 g of CMGs were added and, after the homogenization of all compounds, the mixture was cooled at 5 °C using an ice bath. Afterwards, appropriate amounts of coupling reagent (DCC) and catalyst (DMAP) were introduced into the solution and the temperature was set at 25 °C, initiating the reaction (Steglich esterification) [51]. By-products were retrieved through filtering and the final composite was rinsed with anhydrous methanol prior to drying in the vacuum oven (Fisherbrand™ Isotemp™ Model 281A Vacuum Oven, Pittsburgh, PA, USA) for 48 h. The degree of reinforcement in the final composite materials was determined to be equal to 1 and 3 weight percentage (%wt) of CMGs in the polymeric matrix for both polymer samples (1-PS-*b*-PI-OH and 2-PS-*b*-PI-OH, respectively). The final nanocomposite materials are presented in Scheme S1c (Supplementary Materials).

For clarification reasons, the four (4) final samples were labeled, taking into consideration the initial content of CMGs on each polymeric matrix as follows: 1-PS-*b*-PI-*g-*CMGs 1%, 1-PS-*b*-PI-*g-*CMGs 3%, 2-PS-*b*-PI-*g-*CMGs 1%, and 2-PS-*b*-PI-*g-*CMGs 3%, respectively.

## 3. Results and Discussion

### 3.1. Molecular and Thermal Characterization Results

The total number average molecular weights, the dispersity indices, and the absence of any by-products were determined via SEC, further confirming the successful synthesis of well-defined copolymers. In total, two diblock copolymer samples were synthesized, exhibiting the following molecular characteristics as evident in Table 1, where the number average molecular weights, the dispersity, and the mass and volume fractions are presented. Chromatographs (Figure S1a,b) and ^1^H-NMR spectra (Figure S2a,b) are presented in the Supplementary Materials for each sample respectively.

Further confirmation on the existence of –OH groups in the diblock copolymers was accomplished using FT-IR spectroscopy. As evident in Figure S3a in the Supplementary Materials, both samples exhibited a weak peak in the region of 3500 cm^−1^, which is attributed to the hydroxyl groups at the end of the macromolecular chains. It should be noted that the limited intensity of the peak is assigned to the low –OH group percentage. Additionally, in the region of 650–700 cm^−1^, the bending of the –CH bonds is evident, while at 1500 cm^−1^, the C–C stretching vibration of the aromatic ring in the monomeric unit of polystyrene is obvious and, finally, the stretching vibrations of the C–H, C–C, and =C–H groups are located at 2700–3000 cm^−1^ [52,53]. The FT-IR spectrum of the GMCs is also given in the Supplementary Materials (Figure S3b) in order to verify the existence of the –COOH groups at 1630 cm^−1^ through the complete neutralization of the hydroxyl groups.

TGA studies were performed in order to examine the thermal behavior of the modified graphene oxide, where a trivial weight loss at approximately 100 °C is attributed to the water molecules, while the decomposition of functional groups leads to a second significant weight loss at 200 °C [54]. Finally, the complete decomposition of the graphitic lattice occurred at approximately 485 °C (Figure S4a in the Supplementary Materials). Concerning the final composite, materials the TGA thermographs resulted in higher decomposition temperatures in the case of the final composites when compared with the pristine diblock precursors, indicating increased thermal stability attributed to the covalent bonds between the diblock copolymer matrices and the CMGs (Figure S4b,c in the Supplementary Materials).

**Figure 1:**
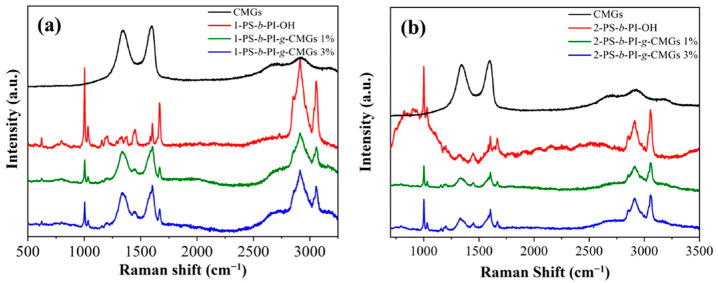
Raman spectra corresponding to (**a**) CMGs (black color), diblock precursor of the 1-PS-*b*-PI-OH type (red color), the final nanocomposites 1-PS-*b*-PI-*g*-CMGs-1% (green color), and 1-PS-*b*-PI-*g*-CMGs-3% (blue color); and (**b**) CMGs (black color), diblock precursor of the 2-PS-*b*-PI-OH type (red color), the final nanocomposites 2-PS-*b*-PI-*g*-CMGs-1% (green color), and 2-PS-*b*-PI-*g*-CMGs-3% (blue color).

Raman spectroscopy was employed in order to determine the structural defects of the CMG sheets calculated from the ratio I_D_/I_G_ (Figure S5 in the Supplementary Materials), as well as the covalent attachment between polymeric chains and CMGs and, consequently, the formation of sp^3^ hybridism. Specifically, at ~1350 cm^−1^ the D band, attributed to the overall structural defects of the CMGs, while at ~1592 cm^−1^ the G band, indicates the permitted phononic transmittance derived from sp^2^ carbon hybridisms. The D Raman band is located in the region between 1250 and 1450 cm^−1^ and is attributed to the sp^3^ carbons, indicating the structural defects (hetero-atoms, grain boundaries, vacancies, and so on), while G band is assigned to the planar vibrations of sp^2^ carbon bonds at approximately 1600 cm^−1^. High-quality samples exhibit a low I_D_/I_G_ ratio, indicating the lack of defects, and thus the enhanced lattice crystallinity degree [55,56]. Raman spectroscopy was applied to all samples, including intermediate diblock copolymers of the PS-*b*-PI-OH type and the final nanocomposites with different concentrations, and the results are presented in Figure 1a,b. Regarding Figure 1a, the black colored line corresponds to the chemically modified graphene oxide, where the D band is evident at 1340 cm^−1^, while the G band is observed at 1580 cm^−1^. The diblock copolymer precursor is presented with red color and the wagging vibrations at 1050–1150 cm^−1^ are assigned to the –CH_2_ and –CH bonds of polystyrene and poly(isoprene), respectively. The intense peak that appeared at 1668 cm^−1^ is ascribed to C=O (stretching). The double carbon bonds [53] (=CH_2_) of the monomeric unit of poly(isoprene) are obvious at 1300 cm^−1^, the band at 1452 cm^–1^ is attributed to (–CH_2_ bending) of the poly(isoprene) segments, while the C=C aromatic bonds in the monomeric unit of the PS are evident at 1580–1600 cm^−1^. Moreover, the intense signal at 1002 cm^−1^ is assigned to C–C aromatic (stretching), while the band at 618 cm^−1^ is imputed to C–H aromatic (stretching out of plane in the opposite direction). Finally, a strong peak at 3058 cm^–1^ is assigned to the aromatic protons (C–H stretching in plane bending), while the bands at 2850–2930 cm^–1^ are attributed to the alkane vibrations (C–H anti-symmetric stretching –CH_3_). Regarding the final composite materials, in addition to the characteristic intensities attributed to the intermediate product, the two characteristic vibrations attributed to the graphitic structures are evident. No substantial differentiations were observed on the intensities when the CMGs content was increased from 1 to 3 wt%. Accordingly in Figure 1b, the coherent intensities can be clearly observed, further verifying the covalent bonding between CMGs and PS-*b*-PI-OH diblock precursors, attributed to the esterification reactions.

**Figure 2:**
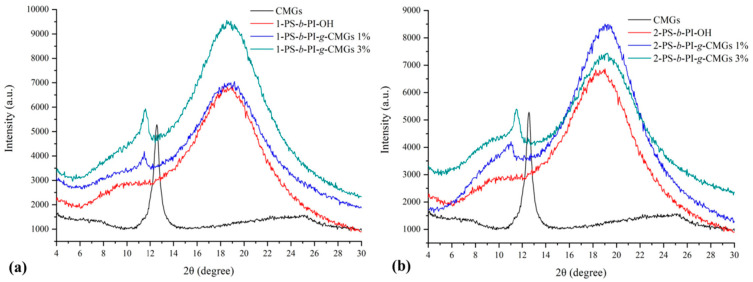
XRD patterns corresponding to (**a**) CMGs (black color), diblock precursor of the 1-PS-*b*-PI-OH type (red color), the final nanocomposites 1-PS-*b*-PI-*g*-CMGs 1% (blue color), and 1-PS-*b*-PI-*g*-CMGs 3% (green color); and (**b**) CMGs (black color), diblock precursor of the 2-PS-*b*-PI-OH type (red color), the final nanocomposites 2-PS-*b*-PI-*g*-CMGs 1% (blue color), and 2-PS-*b*-PI-*g*-CMGs 3% (green color).

X-ray diffraction studies were conducted in order to justify the successful exfoliation of CMG sheets owing to the grafting of the polymeric chains. In Figure 2a, the XRD patterns of CMGs, 1-PS-*b*-PI-OH, 1-PS-*b*-PI-*g*-CMGs 1%, and 1-PS-*b*-PI-*g*-CMGs 3% are illustrated, where the black line corresponds to the CMGs with the characteristic diffraction peak at 2θ° = 12.6° and d-spacing equal to 6.6 Å (distance between two consecutive graphene sheets or interlayer spacing), while the diffraction peaks of 1-PS-*b*-PI-*g*-CMGs 1% and 1-PS-*b*-PI-*g*-CMGs 3% appear at 2θ° = 11.4°, leading to d = 7.3 Å in both cases. Similarly, in Figure 2b, the characteristic diffraction peaks for 2-PS-*b*-PI-*g*-CMGs 1% and 2-PS-*b*-PI-*g*-CMGs 3% are exhibited at 2θ° = 11.0° and 2θ° = 10.4°, leading to d = 7.6 Å and d = 7.3 Å, respectively. Partial polymeric chain interference between consecutive CMG sheets is evident owing to the slight increase of the d-spacing approximately equal to 0.7–1 Å (6.6 Å for the neat CMGs and 7.3 Å or 7.6 Å for the composite materials).

### 3.2. Morphological Characterization

Transition electron microscopy measurements were carried out in order to verify the incorporation of the CMGs into the polymeric matrices, as well as the effect of the incorporated CMGs on the adopted morphology. Both samples were casted in 5% *w/v* solution using a non-selective solvent, namely toluene, in order to obtain equilibrium morphologies regarding the diblock copolymer, for 5–7 days in a saturated environment. It should be mentioned that the dissolution of the diblock copolymers resulted in satisfactory dispersion of the CMGs for longer periods in organic solvents (Figure S6 in the Supplementary Materials). Both samples were submitted to an annealing process above the glass transition temperature of both segments (120 °C) for 48 h. The almost identical electron densities between the PS and PI segments require the utilization of staining processes with aqueous solution of OsO_4_ (Science Services, Munich, Germany) for approximately 2 h, prior to morphological studies [49]. Based on the literature, the adopted morphologies for the specific systems were in accordance with the experimentally obtained results, namely lamellar morphology in the case of 1-PS-*b*-PI-*g*-CMGs 1–3% and hexagonally close packed cylindrical morphology in the case of 2-PS-*b*-PI-*g*-CMGs 1–3%. However, the main purpose of this work was to study the structure/properties relationship of the composite materials and, specifically, the arrangement of the modified graphene sheets when incorporated into the polymeric matrices.

**Figure 3:**
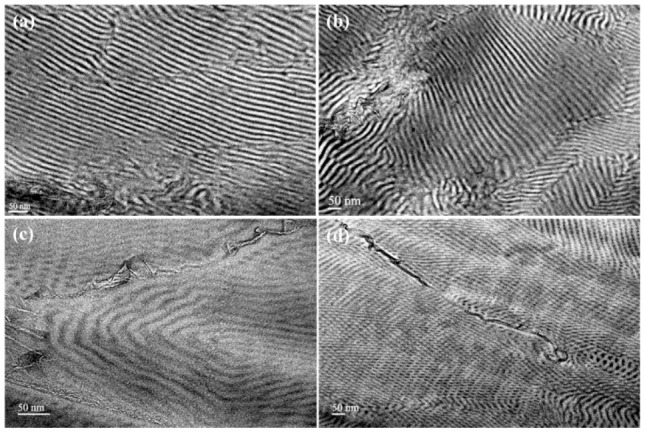
Bright field TEM images corresponding to the (**a**) 1-PS-*b*-PI-*g*-CMGs 1%, (**b**) 1-PS-*b*-PI-*g*-CMGs 3%, (**c**) 2-PS-*b*-PI-*g*-CMGs 1%, and (**d**) 2-PS-*b*-PI-*g*-CMGs 3% final composite materials after thermal annealing at 120 °C for 48 h, sequential microtoming, and staining with vapors of OsO_4_ for approximately 2 h.

In Figure 3a,b, TEM micrographs regarding 1-PS-*b*-PI-*g*-CMGs 1% and 1-PS-*b*-PI-*g*-CMGs 3%, respectively, are presented. Alternating lamellae corresponding to white PS and black PI domains along with CMGs incorporated in the PI lamellar domains attributed to the adopted synthetic route, which included the esterification reaction between hydroxyl (PS-*b*-PI-OH) and carboxyl groups (CMG–COOH). The low content of CMGs in the polymeric matrix during synthesis reaction led to limited incorporation, which is evident in the TEM images, where a minimal amount of CMGs was embedded into the PI segments. For the second case (Figure 3c,d), hexagonally close packed cylinders were obtained for both 2-PS-*b*-PI-*g*-CMGs 1% and 2-PS-*b*-PI-*g*-CMGs 3%, where the PS (white) majority constituted the matrix and the PI (black) minority formed the cylindrical domains. As expected, the arrangement of the CMGs in the cylindrical domains was not favored owing to the inability of the CMGs to curve in the confined cylindrical structure, resulting in graphitic vertical structures evident only in grain boundaries.

## 4. Conclusions

Employing anionic polymerization led to the synthesis of well-defined diblock copolymers of the PS-*b*-PI-OH type, presenting narrow dispersity as well as molecular and structural homogeneity, capable of reacting through esterification with chemically modified graphene sheets, resulting in the final composite materials. The sensitivity of living ends during anionic polymerization renders “*grafting to*” methods for the preparation of composite materials quite challenging, which is further supported by the lack of corresponding published works in the literature. Therefore, in this work, esterification reactions were preferred as a facile method for the incorporation of modified graphitic structures to the diblock copolymer precursors.

The successful loading of the CMGs in the polymeric matrices was supported using various techniques. All samples were molecularly, thermally, and morphologically characterized using SEC, ^1^H-NMR, FT-IR, TGA, Raman spectroscopy, XRD, and TEM. It should be noted that the structure/properties relationships of such systems, prepared through the sequential anionic addition polymerization technique and subsequent esterification reaction, have not yet been reported in the literature. The obtained morphologies of the final nanocomposites showcased microphase separation in the presence of CMGs that were arranged according to the adopted morphologies. Specifically, graphene sheets were incorporated between the alternating lamellar domains, while the inability of graphene sheets to curve within the cylindrical topology led to CMGs’ alignment to the grain boundaries.

## Figures and Tables

**Table 1:** Molecular characterization results for the linear diblock copolymers of the PS-*b*-PI-OH type.

Sample Number	Samples	$\left({\bar{M}}_{n}\right){{\mathrm{PS}}_{\;}}^{a}$ (g/mol)	$\left({\bar{M}}_{n}\right){P_{Ι}}^{\;a}$ (kg/mol)	$\left({\bar{M}}_{n}\right){{\mathrm{Total}}_{\;}}^{a}$ (kg/mol)	*Đ*_total_^a^	*f*_PS_^b^	*φ*_PS_^c^
1	PS-*b*-PI-OH	21,000	17,500	38,500	1.04	0.51	0.52
2	PS-*b*-PI-OH	31,000	10,500	41,500	1.06	0.73	0.74

^a^ SEC in THF at 30 °C, ^b 1^H-NMR Measurements in CDCl_3_ at 25 °C, and ^c^ from the Equation $\varphi_{PS}=\frac{f_{PS}\rho_{PI}}{f_{PS}\rho_{PI}+\left(1-f_{PS}\right)\rho_{PS}}$.

## Data Availability

The data presented in this study are available upon request from the corresponding author.

## Supplementary Materials

The following are available online at https://www.mdpi.com/article/10.3390/polym13142308/s1, Scheme S1: Schematic illustration of the (a) synthesis of the PS-*b*-PI-OH type, (b) synthesis of the carboxylation of the graphene oxide sheets, and (c) synthesis of the final composite materials of the PS-*b*-PI-*g*-CMGs type. Figure S1: SEC chromatographs of the synthesized diblock copolymers corresponding to (a) the PS homopolymer (blue color) and the hydroxyl terminated diblock copolymer of the 1-PS-*b*-PI-OH type (red color) and (b) the PS homopolymer (blue color) and the hydroxyl terminated diblock copolymer of the 2-PS-*b*-PI-OH type (red color). Figure S2: ^1^H-NMR spectra corresponding to (a) 1-PS-*b*-PI-OH and (b) 2-PS-*b*-PI-OH diblock copolymer precursors, where in both cases, the characteristic chemical shift at 3.5 ppm confirms the presence of –OH groups after the successful end-capping reaction using two monomeric units of ethylene oxide. Figure S3: FT-IR spectra of (a) the hydroxyl terminated diblock copolymers of the PS-*b*-PI-OH type and (b) chemically modified graphene. Figure S4: TGA thermographs corresponding to (a) the neat CMGs; (b) 1-PS-*b*-PI-OH (black color), 1-PS-*b*-PI-*g*-CMGs 1% (red color), and 1-PS-*b*-PI-*g*-CMGs 3% (blue color); and (c) 2-PS-*b*-PI-OH (black color), 2-PS-*b*-PI-*g*-CMGs 1% (red color), and 2-PS-*b*-PI-*g*-CMGs 3% (blue color). Figure S5: Raman spectrum corresponding to the chemically modified graphene. Figure S6: Optical observation of the final composite materials dispersed in toluene. From left to right, 1-PS-*b*-PI-*g*-CMGs 1%, 1-PS-*b*-PI-*g*-CMGs 3%, 2-PS-*b*-PI-*g*-CMGs 1%, and 2-PS-*b*-PI-*g*-CMGs 3%.
