# Supplementary material for: Structure/Properties Relationship of Anionically Synthesized Diblock Copolymers “Grafted to” Chemically Modified Graphene
Source: Polymers (Basel). 2021 Jul 14;13(14):2308. doi: 10.3390/polym13142308 (PMC8309249; doi:10.3390/polym13142308)
Supplement: Supplementary file 1 [file polymers-13-02308-s001.zip › polymers-1296910-supplementary.pdf]

Supplementary Materials

Structure/Properties Relationship of Anionically Synthesized Diblock Copolymers "*Grafted to*" Chemically Modified Graphene

Dimitrios Katsigiannopoulos [1], Eftychia Grana [1], Konstantina Tsitoni [1], Ioannis Moutsios [1], Gkreti-Maria Manesi [1], Evgeniia A. Nikitina [2], Nikolaos Chalmpes [1], Dimitrios Moschovas [1,2], Dimitrios Gournis [1], Dimitri A. Ivanov [2,3,4] and Apostolos Avgeropoulos [1,2,*]

[1] Department of Materials Science Engineering, University of Ioannina, University Campus-Dourouti, 45110 Ioannina, Greece; Dimitris.Katsigiannopoulos@gmail.com (D.K.); Eftychia.Grana@gmail.com (E.G.); k.tsitoni@uoi.gr (K.T.); imoutsios@uoi.gr (I.M.); gretimanesi@uoi.gr (G.-M.M.); chalmpesnikos@gmail.com (N.C.); dmoschov@uoi.gr (D.M.); dgourni@uoi.gr (D.G.)
[2] Faculty of Chemistry, Lomonosov Moscow State University (MSU), GSP-1, 1-3 Leninskiye Gory, 119991 Moscow, Russia; nikitina.ea@phystech.edu (E.A.N.); dimitri.ivanov.2014@gmail.com (D.A.I.)
[3] Institute of Problems of Chemical Physics, Russian Academy of Sciences, Chernogolovka, 142432 Moscow, Russia
[4] Institut de Sciences des Matériaux de Mulhouse—IS2M, CNRS UMR7361, 15 Jean Starcky, 68057 Mulhouse, France
* Correspondence: aavger@uoi.gr; Tel.: +30-26-5100-9001

The following data are given in the Supplementary Materials:

(A) Schematic Illustration of the Synthetic Routes

(B) SEC Characterization Chromatographs

(C) ^{1}H-NMR Characterization Spectra

(D) FT-IR Characterization Spectra

(E) TGA Thermal Characterization Thermographs

(F) Raman Spectrum of the Chemically Modified Graphene Sheets (CMGs)

(G) Optical Observation of the final composite materials dispersed in toluene

(A) Schematic Illustration of the Synthetic Routes

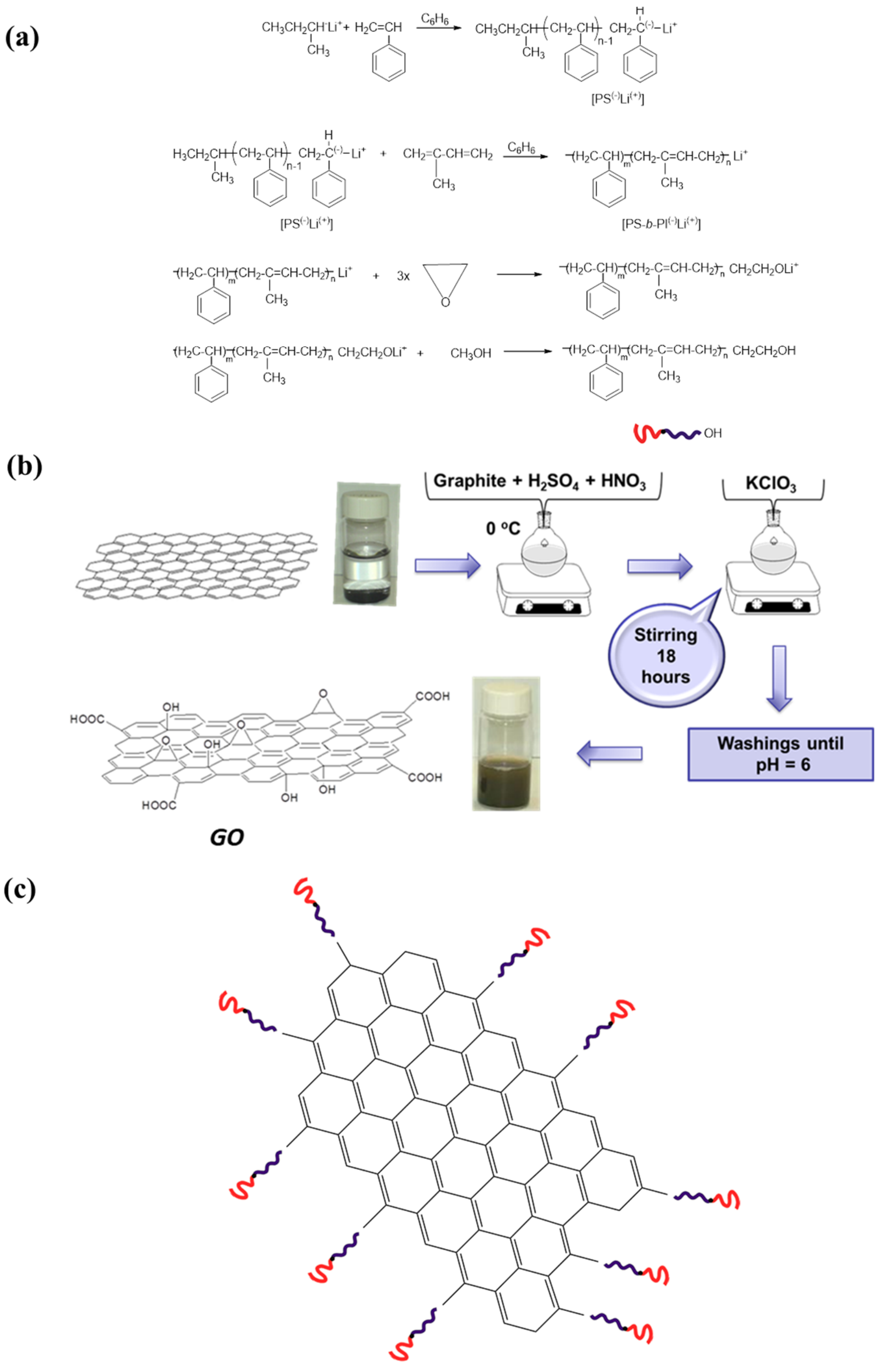

Scheme S1. Schematic illustration of the (**a**) synthesis of the diblock copolymer precursor of the PS-*b*-PI-OH type, (**b**) carboxylation of the graphene oxide sheets and (c) synthesis of the final composite materials of the PS-*b*-PI-*g*-CMGs type.

(B) SEC Characterization Chromatographs

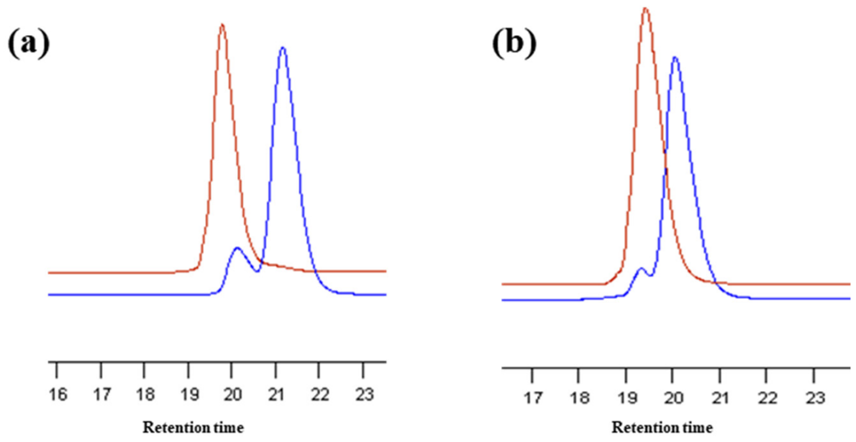

Figure S1. SEC chromatographs of the synthesized diblock copolymers corresponding to: (**a**) the PS homopolymer (blue color) and the hydroxyl terminated diblock copolymer of the 1-PS-*b*-PI-OH type (red color), (**b**) the PS homopolymer (blue color) and the hydroxyl terminated diblock copolymer of the 2-PS-*b*-PI-OH type (red color).

The small peaks on the left at the blue chromatographs (Figure S1a and S1b), are attributed to the termination of the small aliquot obtained during the synthesis procedure. These peaks are not allocated on the final diblock copolymers. They are an outcome of the presence of atmospheric oxygen during the breakage of the aliquot taken while terminating the living chains with methanol, resulting in a slight amount of dimer of the homopolymer.

(C) ^{1}H-NMR Characterization Spectra

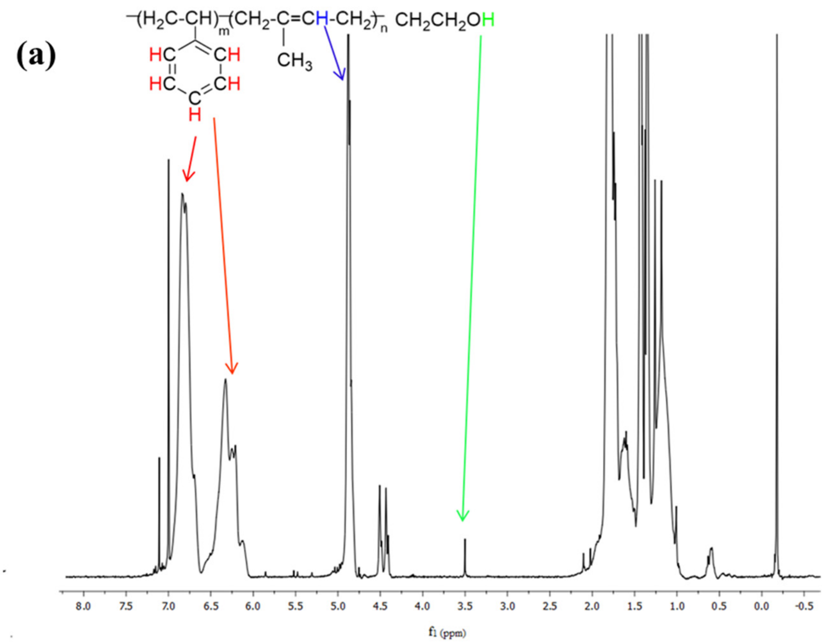

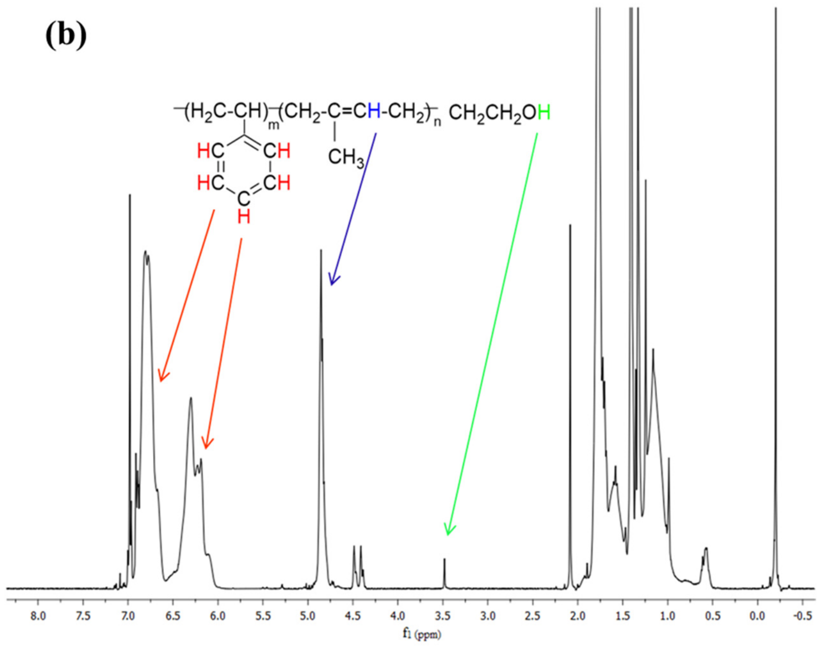

Figure S2. ^{1}H-NMR spectra corresponding to: (**a**) 1-PS-*b*-PI-OH and (**b**) 2-PS-*b*-PI-OH diblock copolymer precursors, where in both cases the characteristic chemical shift at 3.5 ppm confirms the presence of –OH groups after the successful end-capping reaction using two monomeric units of ethylene oxide.

(D) FT-IR Characterization Spectra

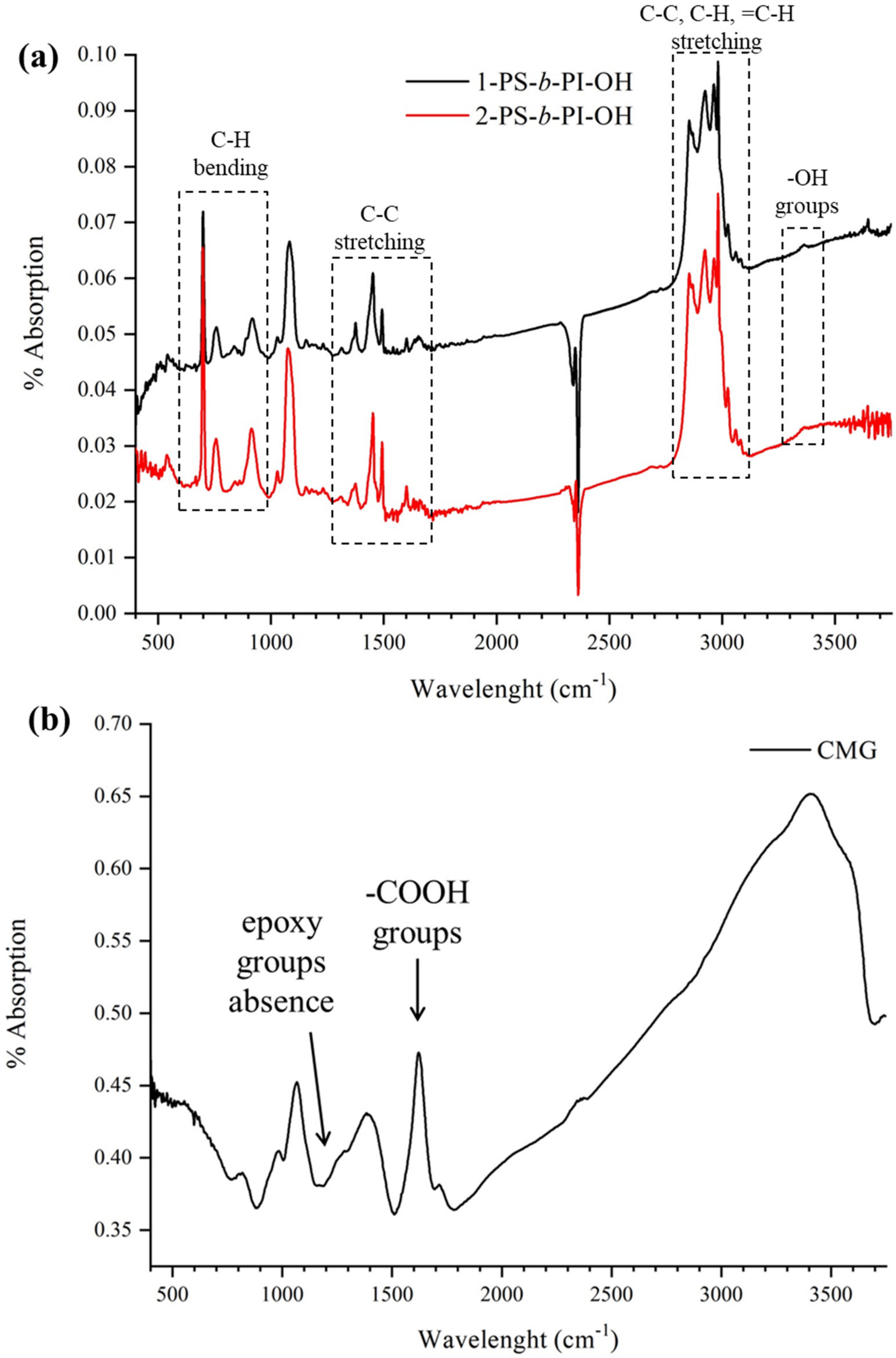

Figure S3. FT-IR spectra of: (**a**) the hydroxyl terminated diblock copolymers of the PS-*b*-PI-OH type and (**b**) chemically modified graphene.

(E) TGA Thermal Characterization Thermographs

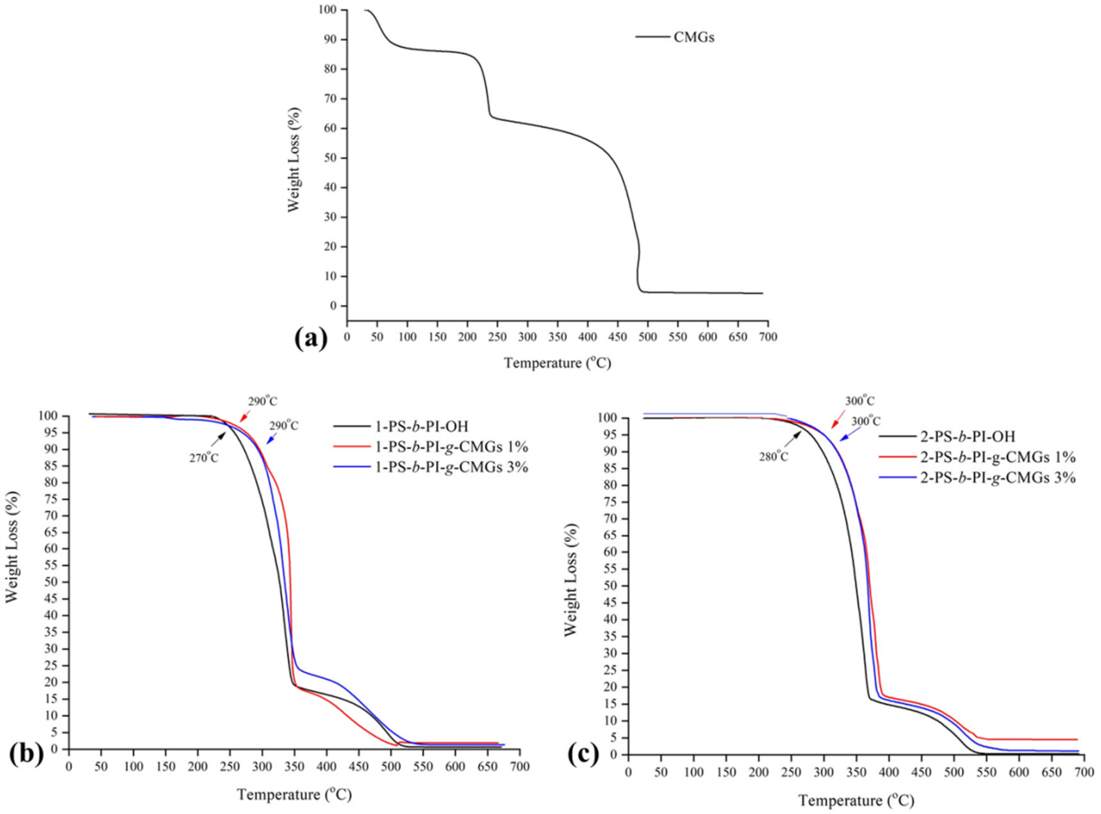

Figure S4. TGA thermographs corresponding to: (**a**) the neat CMGs, (**b**) the 1-PS-*b*-PI-OH (black color), 1-PS-*b*-PI-*g*-CMGs 1% (red color), 1-PS-*b*-PI-*g*-CMGs 3% (blue color) and (**c**) the 2-PS-*b*-PI-OH (black color), 2-PS-*b*-PI-*g*-CMGs 1% (red color), 2-PS-*b*-PI-*g*-CMGs 3% (blue color).

(F) Raman Spectrum of the Chemically Modified Graphene Sheets (CMGs)

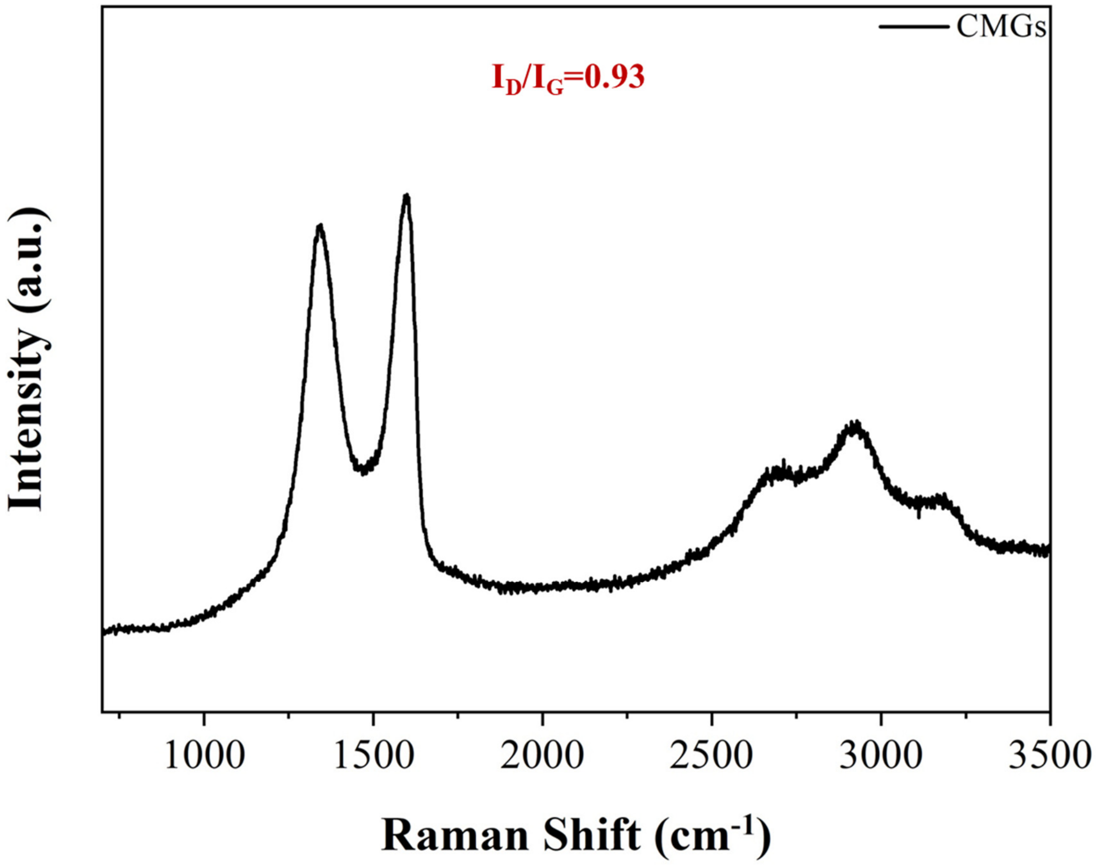

Figure S5. Raman spectrum corresponding to the chemically modified graphene.

(G) Optical Observation of the final composite materials dispersed in toluene

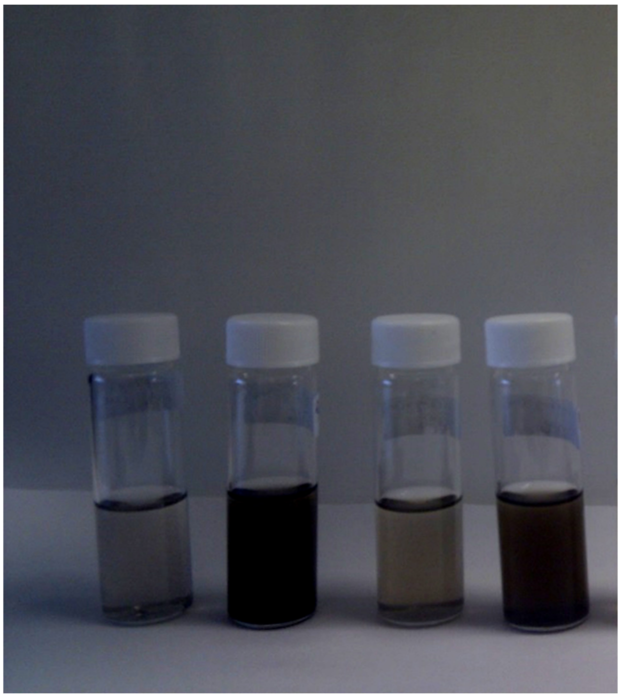

Figure S6. Optical observation of the final composite materials dispersed in toluene. From left to right 1-PS-*b*-PI-*g*-CMGs 1%, 1-PS-*b*-PI-*g*-CMGs 3%, 2-PS-*b*-PI-*g*-CMGs 1% and 2-PS-*b*-PI-*g*-CMGs 3%.
